# Functional Expression of TRPM8 and TRPA1 Channels in Rat Odontoblasts

**DOI:** 10.1371/journal.pone.0082233

**Published:** 2013-12-16

**Authors:** Maki Tsumura, Ubaidus Sobhan, Masaki Sato, Miyuki Shimada, Akihiro Nishiyama, Aya Kawaguchi, Manabu Soya, Hidetaka Kuroda, Masakazu Tazaki, Yoshiyuki Shibukawa

**Affiliations:** 1 Department of Physiology, Tokyo Dental College, Chiba, Japan; 2 Clinical Research Center, National Center for Child Health and Development, Tokyo, Japan; 3 Department of Clinical Oral Health Science, Tokyo Dental College, Tokyo, Japan; 4 Department of Oral Medicine, Oral and Maxillofacial Surgery, Tokyo Dental College, Chiba, Japan; 5 Department of Dental Anesthesiology, Tokyo Dental College, Chiba, Japan; 6 Department of Anesthesiology and Pain Relief Center, University of Tokyo Hospital, Tokyo, Japan; University of Houston, United States of America

## Abstract

Odontoblasts produce dentin during development, throughout life, and in response to pathological conditions by sensing stimulation of exposed dentin. The functional properties and localization patterns of transient receptor potential (TRP) melastatin subfamily member 8 (TRPM8) and ankyrin subfamily member 1 (TRPA1) channels in odontoblasts remain to be clarified. We investigated the localization and the pharmacological, biophysical, and mechano-sensitive properties of TRPM8 and TRPA1 channels in rat odontoblasts. Menthol and icilin increased the intracellular free Ca^2+^ concentration ([Ca^2+^]_i_). Icilin-, WS3-, or WS12-induced [Ca^2+^]_i_ increases were inhibited by capsazepine or 5-benzyloxytriptamine. The increase in [Ca^2+^]_i_ elicited by allyl isothiocyanate (AITC) was inhibited by HC030031. WS12 and AITC exerted a desensitizing effect on [Ca^2+^]_i_ increase. Low-temperature stimuli elicited [Ca^2+^]_i_ increases that are sensitive to both 5-benzyloxytriptamine and HC030031. Hypotonic stimulation-induced membrane stretch increased [Ca^2+^]_i_; HC030031 but not 5-benzyloxytriptamine inhibited the effect. The results suggest that TRPM8 channels in rat odontoblasts play a role in detecting low-temperature stimulation of the dentin surface and that TRPA1 channels are involved in sensing membrane stretching and low-temperature stimulation. The results also indicate that odontoblasts act as mechanical and thermal receptor cells, detecting the stimulation of exposed dentin to drive multiple cellular functions, such as sensory transduction.

## Introduction

Odontoblasts are tall columnar cells that are arranged along the junction between the dentin and dental pulp (the outer surface of the dental pulp faces the inner surface of the dentin) and possess cellular processes that lie inside the dentinal tubules, which are tubular microstructures of dentin. These cells are responsible for dentin formation and mineralization (dentinogenesis) during physiological and developmental processes [1]. Dentinogenesis is also activated by various stimuli applied on the dentin surface, such as mechanical, thermal, pH-related, osmotic, and chemical stimuli [2], [3]. These stimuli also cause dentinal sensitivity in the form of tooth pain. The primary mechanism underlying the generation of dentinal sensitivity has been well documented by the “hydrodynamic theory,” which is based on the microarchitecture of dentin [4]–[6]. The dentin is covered by enamel but is exposed when enamel lesions are formed. The dentin is penetrated by dentinal tubules, which contain dentinal fluid that acts as a hydraulic link between the surface of dentin and the dental-pulp end of the tubules. The intradental afferent-neuron-innervated dental pulp travels a short way into the dentinal tubule along with the odontoblast process. Thus, stimuli applied to the exposed dentin surface elicit fluid flow in dentinal tubules, which in turn induce cellular deformation of nerve endings and odontoblast processes inside the tubules and directly stimulate nerve endings and/or odontoblast processes [4]–[6]. However, the role played by odontoblasts in this sensory transduction sequence as well as in the receptive mechanisms underlying dentin stimulation-induced dentinogenesis remains unclear [5], [6].

Transient receptor potential (TRP) channels, nonselective cation channels constituting 6 main subfamilies, play an important role in nociception and thermo-, osmo-, and mechano-sensation [7], [8]. Recent studies demonstrated that acutely isolated rat and mouse odontoblasts, cultured human dental pulp cells, and cultured mouse odontoblast lineage cells express TRP vanilloid (TRPV) channels. These TRPV channels respond to pH-related, thermal, or hypotonic (membrane stretching) stimulation; extracellular stimulants; or endogenous agonists [5], [9]–[13]. Thus, the expression and the thermal, pharmacological, and mechanical sensitivities of TRPV channel subfamilies in odontoblasts have been well documented. However, the expression and function of TRP melastatin subfamily member 8 (TRPM8) and TRP ankyrin subfamily member 1 (TRPA1) channels in odontoblasts, which are responsive to low-temperature stimuli [14], remain controversial.

El Karim et al. (2011) reported that long-term cultured human odontoblast-like dental pulp cells express TRPM8 and TRPA1 channels. However, the expression of these channels in acutely isolated odontoblasts from rat or mouse has not yet been demonstrated [12], [15]. Thus, the expression patterns of TRPM8 and TRPA1 channels in odontoblasts remain to be elucidated. In addition, although the pharmacological and thermal activation of these channels has been described in cultured dental pulp cells [9], the details of their pharmacological, biophysical and mechano-sensitive properties have not been determined in acutely isolated odontoblasts with intact/native characteristics.

TRPM8 channels are activated by temperatures of 8–28°C or by cooling compounds such as icilin, menthol, eucalyptol, WS3, and WS12 [16]–[18]. Meanwhile, TRPA1 channels are activated by noxious cold temperature (<17°C), mechanical stimulation, or chemical compounds, including menthol, icilin, allyl isothiocyanate (AITC), nicotine, allicin, cinnamaldehyde, and bradykinin [18]–[20]. TRPA1 channels also act as putative transducers of natural physical stimuli, including cold and mechanical force [20], [21]. Endogenous substances such as prostaglandins and nitrated fatty acids also activate these channels [22].

The purpose of this study was to investigate the expression patterns and the pharmacological, biophysical, and mechano-sensitive properties of TRPM8 and TRPA1 channels in acutely isolated rat odontoblasts to clarify whether TRPM8- and/or TRPA1-mediated signal transduction mechanisms are involved in the physiological or pathological stimulation of odontoblasts.

## Materials and Methods

### Ethical Approval

All animals were treated in agreement with the guidelines established by the National Institutes of Health, USA regarding the care and use of animals for experimental procedures. The study was approved by the Ethics Committee of our institute (No. 230303 and No. 240301).

### Immunohistochemistry and Immunofluorescence

Male Wistar rats (150–200 g) were perfused through the left ventricle with 4% paraformaldehyde diluted in 0.1 M phosphate buffer under anesthesia (pentobarbital sodium; 50 mg/kg; KS, Tokyo, Japan). Dissected mandibles were immersed in 4% paraformaldehyde for an additional 12 hr at 4°C. After decalcification with 10% EDTA-2Na solution, selected specimens were dehydrated through a graded ethanol series, embedded in paraffin, and sagittally sectioned to 5 µm in thickness. Dewaxed sections were treated with 0.1% hydrogen peroxide for 15 min and 1 µg/mL trypsin for 30 min. After pre-incubation with 10% goat serum for 30 min at room temperature, sections were incubated with rabbit polyclonal antibody against TRPM8 (catalog No. ab104569; lot No. GR30468-3) or TRPA1 channels (catalog No. ab58844; lot No. GR27084-3) (1∶100, Abcam, Tokyo, Japan) overnight at 4°C. For immunohistochemistry, sections were incubated with HRP-conjugated goat anti-rabbit IgG (1∶100, DAKO Corporation, Glostrup, Denmark) and rinsed with PBS. Immunoreactivity was detected with 3,3′-diaminobenzidine tetrahydrochloride (DAB; Dojindo Laboratories, Kumamoto, Japan). Paraffin-embedded sections were counterstained with methyl green. For immunofluorescence, sections treated with antibody against TRPM8 or TRPA1 channels were incubated with secondary antibody (Alexa Fluor 488 goat anti-rabbit IgG, 1∶100, Invitrogen, Grand Island, NY, USA) at room temperature for 1 hr. To stain nuclei, 4′,6-diamino-2-phenylindole was used at room temperature for 5 min with subsequent washing.

### Dental Pulp Slice Preparation

Dental pulp slice preparations were obtained from newborn Wistar rats (3- to 10-day-old) using a previously described method [13]. Briefly, under pentobarbital sodium anesthesia (25 mg/kg), the mandible was dissected. A hemimandible embedded in alginate impression material was sectioned transversely through the incisor at 500-µm thickness using a standard vibrating tissue slicer (Dosaka EM, Kyoto, Japan). A section of mandible was sliced to the required level where the dentin and enamel were directly visible between the bone tissues and dental pulp. The surrounding impression material, bone tissue, enamel, and dentin were removed from the section of mandible under a stereoscopic microscope, and the remaining dental pulp slice was obtained. For this purpose, we selected mandible sections with thin dentin (but with enamel and dentin distinguishable under the microscope) to avoid cellular damage in odontoblasts. Pulp slices were treated with a standard solution containing 0.03% trypsin and 0.17% collagenase at 37°C for 30 min. For [Ca^2+^]_i_ measurement, enzymatically treated and isolated odontoblasts in the dental pulp slice were plated onto a culture dish, immersed in alpha-minimum essential medium (Invitrogen) containing 10% fetal bovine serum and 5% horse serum, and maintained at 37°C in a 5% CO_2_ incubator. The primary cultured odontoblasts in the dental pulp slice were used for [Ca^2+^]_i_ measurement within 24 hr after isolation. These cells were positive for the odontoblast markers dentin matrix protein-1, dentin sialoprotein, and nestin [13] (not shown) and were thus odontoblasts.

### Measurements of Ca^2+^-Sensitive Dye Fluorescence

Cells in dental pulp slices were loaded for 30 min at 37°C in standard solution containing 10 µM fura-2-acetoxymethyl ester (Dojindo Laboratories) and 0.1% (w/v) pluronic acid F-127 (Invitrogen). They were then rinsed with fresh standard solution. A dish with fura-2-loaded odontoblasts was mounted on the stage of a microscope (Olympus, Tokyo, Japan), which was equipped with an Aquacosmos system (Hamamatsu Photonic, Shizuoka, Japan), excitation wavelength selector, and intensified charge-coupled device camera system. Fura-2 fluorescence emission was measured at 510 nm under alternating excitation wavelengths of 380 nm (F380) and 340 nm (F340). [Ca^2+^]_i_ was expressed as the fluorescence ratio (R_F340/F380_) at these two excitation wavelengths, and then expressed as *F/F_0_* units; the R_F340/F380_ value (*F*) was normalized to the resting value (*F/F_0_* = 1.0) in the presence of extracellular Ca^2+^ (*F_0_*).

### Solutions and Reagents

Standard solution containing (in mM) 136 NaCl, 5 KCl, 2.5 (or 0) CaCl_2_, 0.5 MgCl_2_, 10 HEPES, 10 glucose, and 12 NaHCO_3_ (pH 7.4 by Tris) was used as an extracellular solution. Isotonic solution was prepared containing (in mM) 36 NaCl, 200 mannitol, 5 KCl, 2.5 CaCl_2_, 0.5 MgCl_2_, 10 HEPES, 10 glucose, and 12 NaHCO_3_ (pH 7.4 by Tris). To induce membrane stretch [11], hypotonic solution (200 mOsm/L) was prepared with the concentration of mannitol reduced to 64 mM. WS3, WS12, and icilin were obtained from TOCRIS Cookson (Bristol, UK). Allyl isothiocyanate (AITC), 5-benzyloxytryptamine hydrochloride (5-BOT), and trypsin were obtained from Wako Pure Chemical Industries Ltd. (Osaka, Japan). All other reagents were obtained from Sigma Chemical Co. (St Louis, MO, USA). A stock solution of AITC was prepared in ethanol. All other stock solutions were prepared in dimethyl sulfoxide. Stock solutions were diluted to the appropriate concentration with standard or hypotonic solution.

### Statistics

Data are shown as the mean ± standard error (SE) of the mean of *N* observations, where *N* represents the number of experiments. The Wilcoxon t-test or Friedman test and Dunn’s post-hoc test were used to determine the non-parametric statistical significance. A *P*-value <0.05 was considered significant.

## Results

### Localization and Distribution of TRPM8 Channels in Odontoblasts

**Figure 1:**
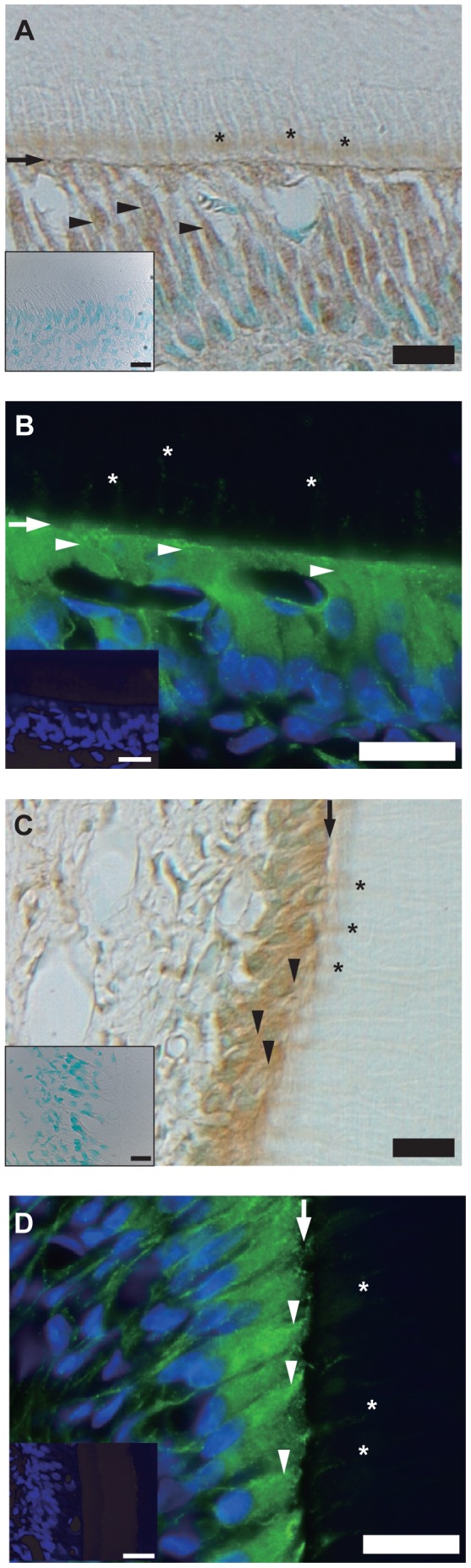
Localization and distribution of TRPM8 channels in odontoblasts. (**A–D**) Tall, columnar, and polarized secretory odontoblasts positive for TRPM8 channel immunoreactivity (brown and green) were observed at dental pulp section in the mandibular incisor (**A and B**) and molar (**C and D**) by immunohistochemical (**A and** **C**) and immunofluorescence (**B** **and D**) analysis. Immunoreactivity was also observed in mature odontoblasts and those obtained from incisors and molars of 16-week-old rats (not shown). TRPM8 channels were localized across entire cell membrane. Arrowheads indicate distal portion of membrane, and asterisks show cellular processes inside dentinal tubules of odontoblasts. Nuclei are shown in blue. Arrows: dentopulpal junction. Scale bars: 20 µm. No fluorescence was detected in negative controls (insets of **A–D**).

Immunohistochemical (brown; Figures 1A and 1C) and immunofluorescence (green; Figures 1B and 1D) observations revealed the expression of TRPM8 channels in tall, columnar rat odontoblasts in sections prepared from mandibular incisors (Figures 1A and 1B) and molars (Figures 1C and 1D). Distinct immunoreactivity for TRPM8 channels was observed across the plasma membrane in odontoblasts.

### [Ca^2+^]_i_ Increase by TRPM8 Channel Agonists

**Figure 2:**
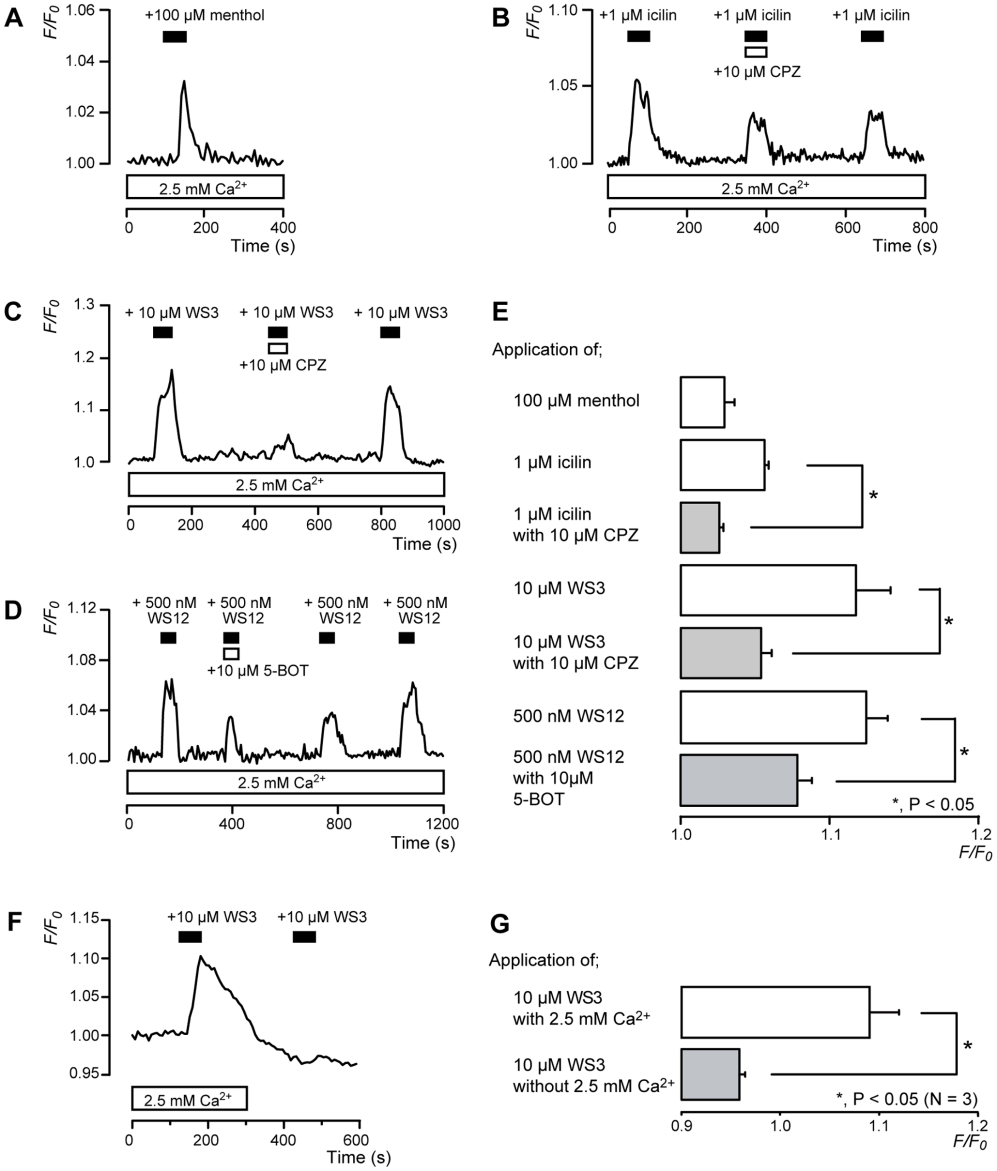
[Ca^2+^]_i_ increase induced by a TRPM8 channel agonists. (**A–D**) Representative traces of transient [Ca^2+^]_i_ increase in response to 100 µM menthol (**A**), 1 µM icilin (**B**), 10 µM WS3 (**C**), or 500 nM WS12 (**D**) in presence of extracellular Ca^2+^ (white boxes at bottom). Addition of 10 µM CPZ (**B** **and C**) or 5-BOT (**D**) inhibited [Ca^2+^]_i_ increase. Bars in each figure indicate when agonists (black) or antagonists (white) were added to external solution. (**E**) Summary bar graphs show increase in [Ca^2+^]_i_ by agonists with (gray columns) or without (open columns) antagonists. Agonists and antagonists used are denoted on left side of graph. (**F and G**) Example trace of transient [Ca^2+^]_i_ increase in response to 10 µM WS3 with (white box at bottom) or without extracellular Ca^2+^ (**F**). Black bars denote when WS3 was added to external solution. Summary bar graphs of [Ca^2+^]_i_ increase without (gray column) or with (open column) extracellular Ca^2+^. Resting value is shown as *F/F_0_* = 1.0. Each bar (in **E** **and** **G**) indicates mean ± SE of several experiments (see text). Statistically significant differences between columns (shown by solid lines) are denoted by asterisks: *P<0.05.

Odontoblasts localized on the rim of the primary cultured dental pulp slices were loaded with fura-2, and [Ca^2+^]_i_ was measured [23]–[26]. Menthol (100 µM; Figure 2A) and icilin (1 µM; Figure 2B), TRPM8 and TRPA1 channel agonists, increased [Ca^2+^]_i_ to peak values of 1.03±0.01 *F/F_0_* units (*N* = 4) and 1.07±0.003 *F/F_0_* units (*N* = 3), respectively (Figure 2E). To activate TRPM8 channels specifically, WS3 and WS12 were used as TRPM8-specific agonists. WS3 (10 µM) increased [Ca^2+^]_i_ to a peak value of 1.12±0.02 *F/F_0_* units (*N* = 4) (Figures 2C and 2E). Both icilin- and WS3-induced [Ca^2+^]_i_ increases were inhibited by 10 µM capsazepine (CPZ), a non-specific TRPM8 channel antagonist (Figures 2B, 2C, and 2E). In addition, WS12 also increased [Ca^2+^]_i_ to a peak value of 1.12±0.01 *F/F_0_* units (*N* = 3). However, 5-benzyloxytriptamine hydrochloride, a specific TRPM8 channel antagonist, reversibly inhibited this increase (Figures 2D and 2E). To examine the pathway of [Ca^2+^]_i_ increase, extracellular Ca^2+^ was removed (Ca^2+^ free solution) during the activation of TRPM8 channels. In the presence of extracellular Ca^2+^, WS3 (10 µM) increased [Ca^2+^]_i_ to 1.09±0.03 *F/F_0_* units (*N* = 3). In the absence of extracellular Ca^2+^, however, no WS3-induced increase in [Ca^2+^]_i_ was observed. The peak value of [Ca^2+^]_i_ in the application of WS3 was 0.96±0.01 *F/F_0_* units (*N* = 3) (Figures 2F and 2G).

### Localization and Distribution of TRPA1 Channels

**Figure 3:**
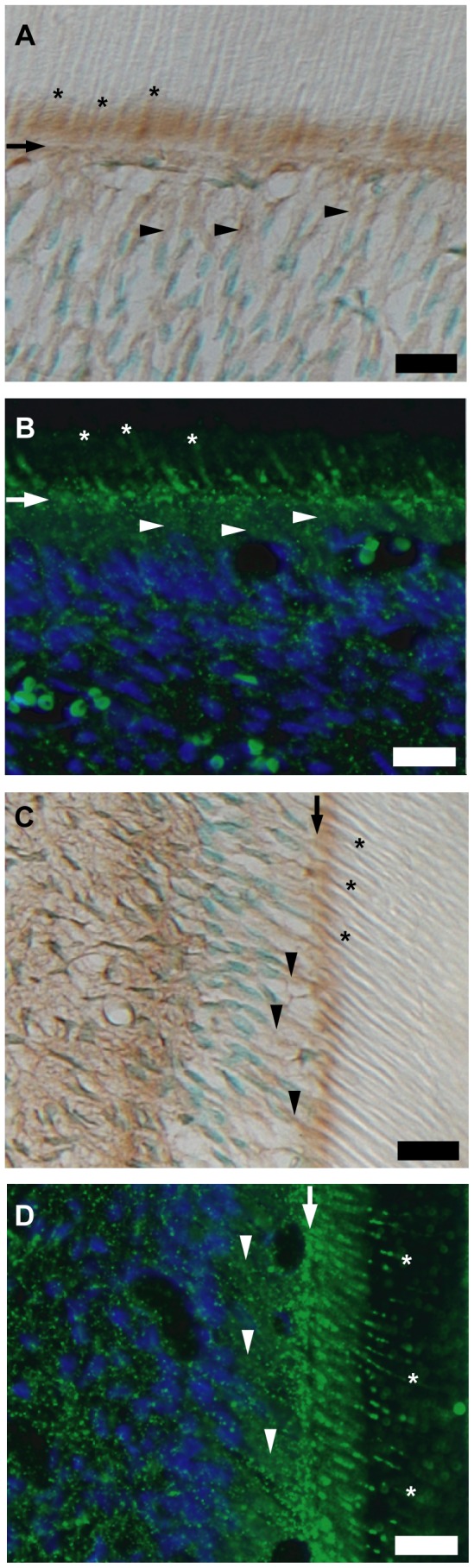
Localization and distribution of TRPA1 channels. (**A–D**) Tall, columnar, and polarized secretory odontoblasts were positive for TRPA1 channel immunoreactivity. Construction of **A–D** is same as in Fig. 1. Although TRPA1 channels were localized on entire cell membrane, intense immunoreactivity was observed on distal membrane of odontoblasts and cellular processes inside dentinal tubules.

Immunoreactivity against TRPA1 channels was observed in odontoblasts; it localized to the distal membrane and the cellular processes of odontoblasts inside dentinal tubules in sections prepared from incisors (brown in Figure 3A; green in Figure 3B) and molars (brown in Figure 3C; green in Figure 3D).

### [Ca^2+^]_i_ Increase by TRPA1 Channel Agonists

**Figure 4:**
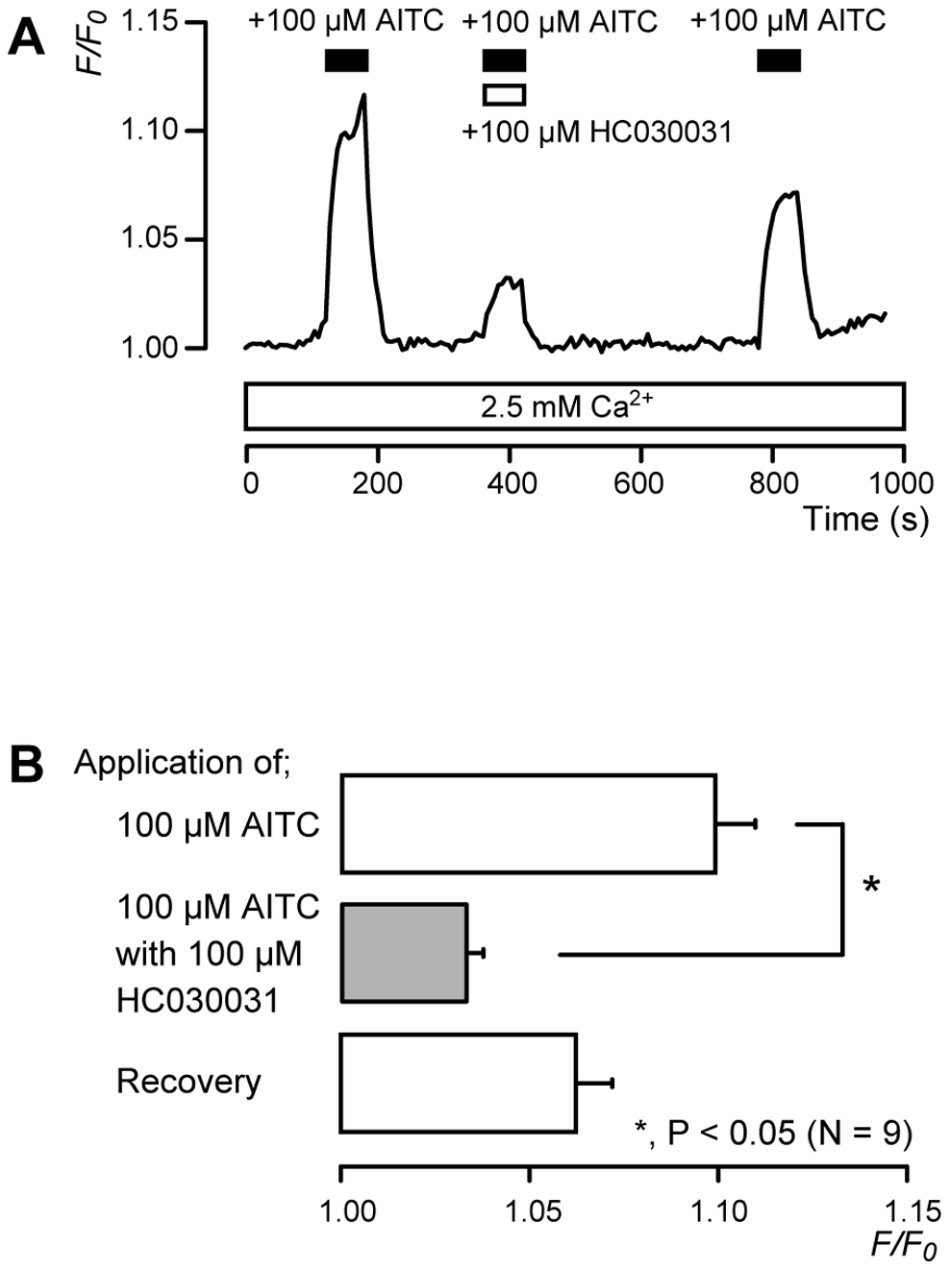
[Ca^2+^]_i_ increase induced by a TRPA1 channel agonist. (**A**) Representative traces of TRPA1-mediated transient [Ca^2+^]_i_ increase in response to 100 µM AITC, with or without 100 µM HC030031, in presence of extracellular Ca^2+^ (white box at bottom). Bars indicate when AITC (black) and HC030031 (white) were added to external solution. (**B**) Summary bar graphs of increase in [Ca^2+^]_i_ in response to 100 µM AITC with (gray column) or without (open columns) 100 µM HC030031. Each bar indicates mean ± SE of 9 experiments. The AITC-induced [Ca^2+^]_i_ increase recovered after removal of HC030031; however, this effect was not significant. This was likely due to the desensitizing effect on the AITC-induced [Ca^2+^]_i_ increase (Figure 5). Statistically significant differences between columns (shown by solid lines) are denoted by asterisks: *P<0.05.

In the presence of extracellular Ca^2+^, 100 µM AITC, a TRPA1 channel agonist, transiently increased [Ca^2+^]_i_ to a peak value of 1.10±0.01 *F/F_0_* units (*N* = 9). The increase was reversibly inhibited by 100 µM HC030031, a TRPA1 channel-specific antagonist, to 1.03±0.004 *F/F_0_* units (*N* = 9) (Figures 4A and 4B).

### Desensitization of TRPM8- or TRPA1-mediated Ca^2+^ Entry by Repeated Application of WS12 or AITC

**Figure 5:**
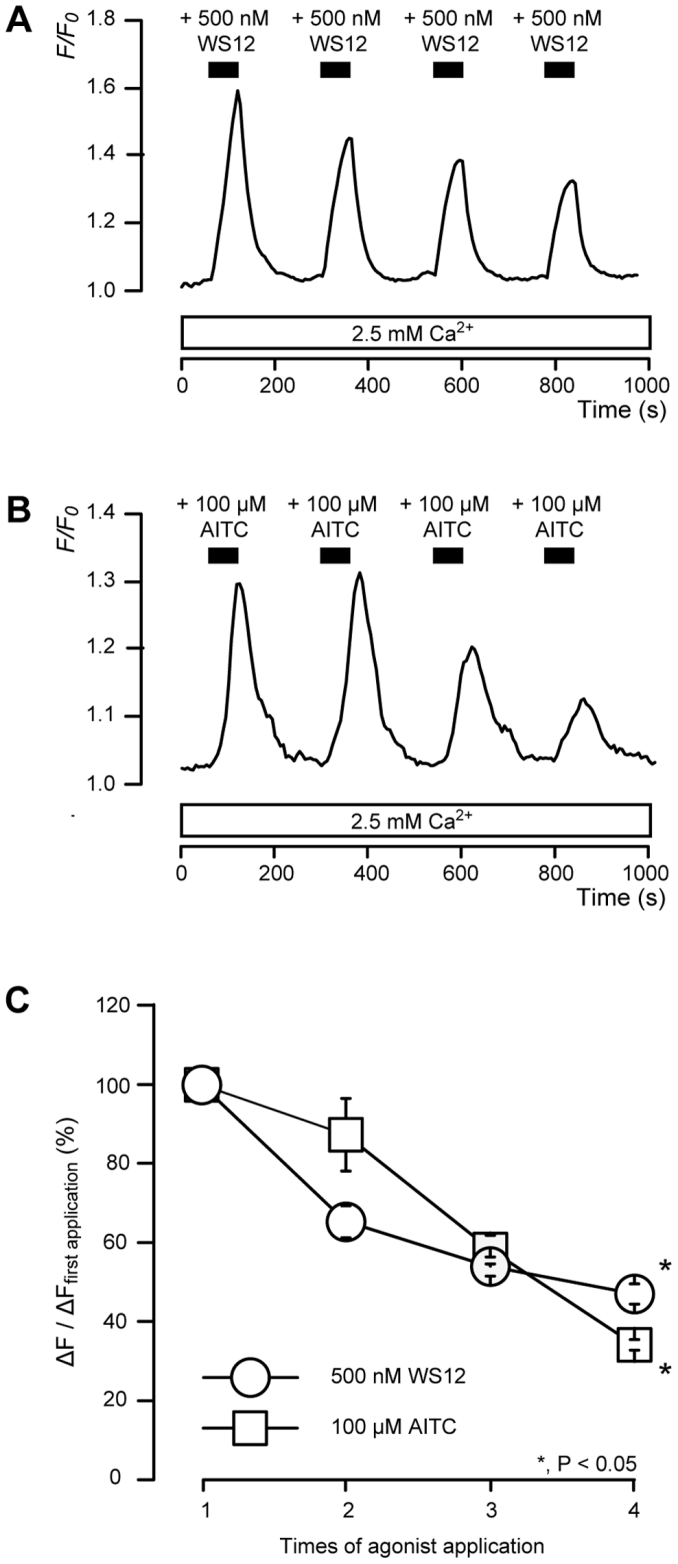
Desensitization of TRPM8 or TRPA1 channels by repeated application of WS12 or AITC. (**A and B**) Representative traces of transient [Ca^2+^]_i_ increase in response to 4 successive applications of 500 nM WS12 (**A**) or 100 µM AITC (**B**) in presence of extracellular Ca^2+^ (white boxes at bottom). Black bars indicate when WS12 or AITC were added (for 1 min) at 3-min intervals. (**C**) Summary graphs of desensitizing effect of WS12 (circles) or AITC (squares). Changes in fluorescence were quantified using following equation: Change in fluorescence (Δ*F*) = *F*–*F_0_*, where Δ*F* upon application of each agonist was normalized to that of first application (100% as control; Δ*F*/Δ*F*_first application_). Each bar indicates mean ± SE of several experiments (see text). Statistically significant differences between data points and between data from first application are denoted by asterisks: *P<0.05.

TRPM8 and TRPA1 channels exhibit Ca^2+^-dependent desensitization [19], [27]. A series of 1-min applications of WS12 (500 nM; Figure 5A) or AITC (100 µM; Figure 5B) at 3-min intervals elicited a significant desensitizing effect on Ca^2+^ entry by the fourth application (Figure 5C). In the presence of extracellular Ca^2+^, the increase in [Ca^2+^]_i_ after the second application of WS12 or AITC was 65.4±2.4% (N = 4) or 87.4±9.2% (*N* = 3), respectively, of the peak value of increase in [Ca^2+^]_i_ after the first application. Additionally, the increase in [Ca^2+^]_i_ after the fourth application of WS12 or AITC was 47.1±2.5% (*N* = 4) or 34.3±1.3% (*N* = 3), respectively, of the increase in [Ca^2+^]_i_ after the first application (Figure 5C).

### Thermal Sensitivity of TRPM8 and TRPA1 Channels

**Figure 6:**
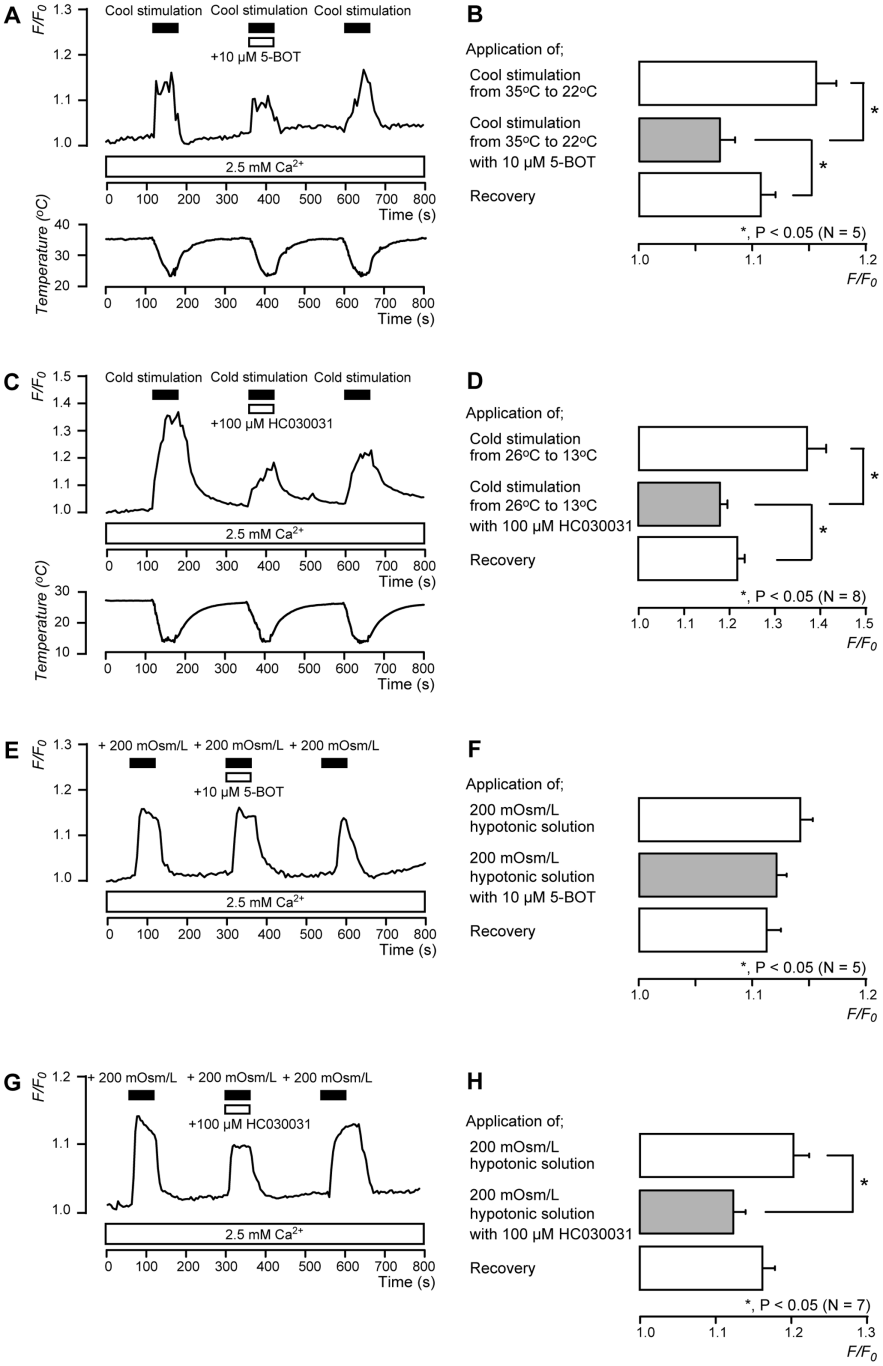
Thermal and membrane stretch sensitivity of TRPM8 and TRPA1 channels. (**A and C**) Representative traces of [Ca^2+^]_i_ increase in response to cool-stimulation from 35°C to 22±1°C or cold-stimulation from 26°C to 13±1°C, with or without 10 µM 5-BOT (**A**) or 100 µM HC030031 (**C**), in presence of extracellular Ca^2+^ (white boxes at bottom). Black bars indicate when cool- or cold-stimulation was applied, and white bars show application of 5-BOT (**A**) or HC030031 (**C**) to external solution. (**B**) Summary bar graphs of increase in [Ca^2+^]_i_ upon cool-stimulation with (gray column) or without (open columns) 10 µM 5-BOT. (**D**) Summary bar graphs of increase in [Ca^2+^]_i_ upon cold-stimulation with (gray column) or without (open columns) 100 µM HC030031. Each bar indicates mean ± SE of 5 (**B**) and 8 (**D**) experiments. Statistically significant differences between columns (shown by solid lines) are denoted by asterisks: *P<0.05. (**E and G**) Example traces of transient [Ca^2+^]_i_ increase in response to 200 mOsm/L hypotonic solution with or without 10 µM 5-BOT (**E**) or 100 µM HC030031 (**G**) in presence of extracellular Ca^2+^ (white boxes at bottom). Bars denote when hypotonic solution (black) and 5-BOT (**E**) or HC030031 (**G**) (white) were added to external solution. (**F and H**) Summary bar graphs of increase in [Ca^2+^]_i_ upon addition of 200 mOsm/L hypotonic solution without (open columns in **F** and **H**) or with (gray column in **F** **and** **H**) 5-BOT or HC030031. Each bar indicates mean ± SE of several experiments (see text). Statistically significant differences between columns (shown by solid lines) are denoted by asterisks: *P<0.05.

To investigate the thermal sensitivity of TRPM8 and TRPA1 channels, we examined the effects of cool- and cold-stimulation on [Ca^2+^]_i_ in the presence of extracellular Ca^2+^. When the temperature of the extracellular solution was reduced from 35 to 22°C as cool-stimulation, [Ca^2+^]_i_ increased. The increase was inhibited by 10 µM 5-BOT (*N* = 5) (Figures 6A and 6B). To activate TRPA1 channels, cold-stimulation was applied by reducing the temperature of the extracellular solution from 26 to 13°C. In response, [Ca^2+^]_i_ increased (*N* = 8). The increase was inhibited by 100 µM HC030031 (*N* = 8) (Figures 6C and 6D).

### TRPA1 Channels, but not TRPM8 Channels, Contribute to Mechano-sensitivity in Odontoblasts

To determine whether TRPM8 and/or TRPA1 channels contributed to mechano-sensitivity, we examined membrane stretch-induced [Ca^2+^]_i_ increase in rat odontoblasts by applying hypotonic solution (200 mOsm/L) [11]. In the presence of extracellular Ca^2+^, the application of hypotonic solution increased [Ca^2+^]_i_ (Figures 6E and 6G). This increase was inhibited significantly by 100 µM HC030031 (*N* = 7) (Figures 6G and 6H); no effect was observed with 10 µM 5-BOT (*N* = 5) (Figures 6E and 6F).

## Discussion

We investigated the expression, localization, and the pharmacological, biophysical, and mechano-sensitive properties of TRPM8 and TRPA1 channels in odontoblasts. Selective TRPM8 channel agonists WS3 or WS12 increased [Ca^2+^]_i_; the increase was inhibited by non-specific (capsazepine) or selective (5-BOT) antagonists of TRPM8 channels. The WS3-induced increase in [Ca^2+^]_i_ was not observed in the absence of extracellular Ca^2+^, indicating that the increase in [Ca^2+^]_i_ upon activation of TRPM8 channels was mediated by Ca^2+^ influx from the extracellular medium. The results also showed that rat odontoblasts were sensitive to AITC and that the AITC-induced [Ca^2+^]_i_ increase was inhibited by the selective TRPA1 antagonist HC030031. These results clearly indicate that rat odontoblasts functionally express TRPM8 and TRPA1 channels.

In the present study, TRPM8 and TRPA1 channels showed sensitivity to low-temperature stimuli (Figure 6). Meanwhile, the membrane stretch-induced increase in [Ca^2+^]_i_ was sensitive to HC030031 but not 5-BOT. The results suggest that TRPA1 channels contribute to thermal- and mechano-sensitivity; TRPM8 channels, however, are not involved in the mechano-sensitive process in odontoblasts. The results are also consistent with data showing intense immunoreactivity for TRPM8 across the cell body and immunoreactivity for TRPA1 on the distal membrane and cellular processes of odontoblasts inside dentinal tubules.

According to the hydrodynamic theory [28] of dentinal sensitivity, exposure of the dentin surface to thermal, osmotic, mechanical, or chemical stimuli results in the movement of fluid in the dentinal tubules, which elicits stretching and/or shear stress at the plasma membrane inside the tubules (i.e., odontoblast processes and/or free nerve endings) [6]. We previously reported that stretching of the odontoblast membrane activates Ca^2+^ entry via TRPV1, TRPV2, and TRPV4 channels [11]. The findings of the current study suggest that TRPA1 channels also detect mechanical stimulation applied to odontoblasts, which is induced by dentinal fluid displacement, by localizing on cellular processes.

The latencies of dentinal sensory and pulpal neuron responses to various stimulations, such as thermal stimulation applied to the dentinal surface, are too short. This indicates that the sensory transduction mechanism in dentin is unlikely to be sensitive to temperature changes inside the dental pulp or at the pulp/dentin interface, where odontoblasts and free nerve endings are located [29], since temperature change is observed at the pulp/dentin interface after a long latency period (around 5 sec) [30]. Therefore, dentinal pain with a short latency (within 1 sec) following various stimuli, including thermal, chemical, osmotic, and mechanical, on the dentin surface results from dentinal fluid movement [6], [29], [31]. This suggests that mechanosensors such as TRPV1, TRPV2, and TRPV4 [11], [13] as well as TRPA1 channels in odontoblasts detecting movement of dentinal fluid may be necessary for dentinal sensation [6], [29], [31].

However, the results from a recent study in humans suggested that pain produced by cold stimuli was due not to a hydrodynamic mechanism, but to something another mechanism such as cold-sensitive molecular sensors located on the odontoblast membrane [32]. Therefore, based on our results and those of previous studies, TRPA1 channels may act as mechanosensors by detecting mechanical stimulation applied to odontoblasts, which are induced by dentinal fluid displacement, while both TRPM8 and TRPA1 channels may be candidates as sensors the generating dentinal sensation after a long latency following cool- and/or cold-stimuli applied to external dentin.

The intradental afferent and its nerve endings reach the odontoblast region and form a dense network of sensory axons (known as the plexus of Raschkow). It was recently suggested that chemical substance(s), such as nitric oxide, ATP, or galanin, may participate in the interaction between odontoblasts and sensory neurons as neurotransmitter(s) [5]. We previously observed, however, that TRP channels act as mechanosensor proteins that activate the release of transmitter(s) from odontoblasts. Released transmitter into the extracellular medium then activates its receptors on trigeminal ganglion neurons (personal communication by YS). Therefore, TRPM8 and TRPA1 channels play important roles in thermal/mechanical sensory transduction mechanisms in odontoblasts.

In conclusion, we demonstrated the expression of TRPM8 and TRPA1 channels in odontoblasts. The results indicate that TRPM8 channels play an important role in detecting low-temperature stimuli, while TRPA1 channels are involved in sensing low-temperature stimulation and plasma membrane mechanical deformation. The results also indicate that odontoblasts act as mechanoreceptor cells and thermal receptor cells, detecting the stimulation of exposed dentin to drive various cellular functions, such as sensory transduction.
